# High Reinfection Rate after Preventive Chemotherapy for Fishborne Zoonotic Trematodes in Vietnam

**DOI:** 10.1371/journal.pntd.0002958

**Published:** 2014-06-19

**Authors:** Tore Lier, Dung Trung Do, Maria Vang Johansen, Thi Hop Nguyen, Anders Dalsgaard, Anne Mette Asfeldt

**Affiliations:** 1 Department for Microbiology and Infection Control, University Hospital of North Norway, Tromsø, Norway; 2 Department for Parasitology, National Institute of Malariology, Parasitology and Entomology (NIMPE), Hanoi, Vietnam; 3 Department of Veterinary Disease Biology, Faculty of Health and Medical Sciences, University of Copenhagen, Copenhagen, Denmark; National Institute of Parasitic Diseases, China

## Abstract

**Background:**

The World Health Organization aims for complete morbidity control of fishborne zoonotic trematodes (FZT) in endemic areas by 2020. The main intervention tool for achieving this goal is regular use of preventive chemotherapy by offering praziquantel to those at risk in endemic areas. The purpose of this study was to investigate the effectiveness of preventive chemotherapy to control FZT in an endemic area in Northern Vietnam.

**Methodology and principle findings:**

We followed a cohort of 396 people who fulfilled the criteria for receiving preventive chemotherapy. Stool samples were examined by Kato-Katz technique for the presence of trematode eggs before, and two, 16, 29 and 60 weeks after preventive chemotherapy. The prevalence of trematode eggs in stool was 40.2% before, 2.3% two weeks after and increased to a cumulative prevalence of 29.8% sixty weeks after preventive chemotherapy.

**Conclusions:**

The effectiveness of preventive chemotherapy as a main component in control of FZT is not well documented in most endemic areas. We found a high reinfection rate within the first year after preventive chemotherapy. Since these trematodes are zoonoses, preventive chemotherapy may not have sufficient impact alone on the transmission to have a lasting effect on the prevalence. Animal reservoirs and farm management practices must be targeted to achieve sustainable control of fishborne zoonotic trematode infections, hence control programs should consider a One Health approach.

## Author Summary

Fishborne zoonotic trematodes (FZT) are small parasitic flatworms (flukes) living either in the intrahepatic bile ducts or in the intestines of humans and many species of animals and birds. Transmission occurs by eating infected raw freshwater fish. FZT are especially common in Southeast and East Asia. WHO suggests using preventive chemotherapy in the control of these parasites by providing an effective drug, praziquantel, once a year to either all inhabitants in the endemic areas or selectively to those who are at special risk because they eat raw fish. Preventive chemotherapy has been used for some years in Northern Vietnam, but the effectiveness has not been thoroughly examined. We followed a group of 396 people who had eaten raw fish and examined the stool for the presence of trematode eggs before preventive chemotherapy and two, 16, 29 and 60 weeks after. The proportions of egg positive persons were 40.2% before, 2.3% two weeks after and increased to 29.8% sixty weeks after preventive chemotherapy. We found a high rate of reinfection. Contribution from animals to the transmission implies that preventive chemotherapy of humans alone will most likely be insufficient to control the FZT infections.

## Introduction

Foodborne trematodiasis is a group of Neglected Tropical Diseases with a considerable impact on human health, especially in Southeast and East Asia. An estimated 56 million people are infected with foodborne trematodes, of which approximately half is due to fishborne zoonotic trematodes (FZT) [1]. FZT infects animals and humans after consumption of raw or insufficiently cooked freshwater fish, in the case of humans often as a traditional dish [2]. Infections with FZT are believed to be emerging, and the rapid growth in aquaculture and consumption of fish raised in endemic areas has been proposed to contribute to infection [3]. FZT include the liver flukes *Clonorchis sinensis* and *Opisthorchis viverrini* as well as a large number of minute intestinal fluke (MIF) species. The prevalence of FZT often shows great regional differences within a particular country. There is also variation in predominance of species within specific geographical areas [4], [5]. The morphological characteristics of eggs belonging to different species are difficult to differentiate by microscopy, and this may lead to unreliable data of their epidemiological distribution [6]. In Northern Vietnam, there is a mix of *C. sinensis* and MIF, especially *Haplorchis* spp., in humans [7], [8]. Studies from the same area have revealed high prevalence of different MIF-species, especially *Haplorchis* spp., in dogs, cats and pigs, suggesting that domestic animals could play an important role as reservoir hosts and in the transmission of FZT [9], [10].

The adult liver flukes enter the intrahepatic bile ducts, while the adult MIF remain in the small intestine. The most serious health hazard associated with liver fluke infection is the increased risk of cholangiocarcinoma. The MIF infections have been less studied, but the clinical manifestations may be less serious and MIF are not considered carcinogenic [5]. The morbidity of the liver trematodes, especially the predisposition for cholangiocarcinoma, is the main reason for establishing control measures [11]. WHO recommends preventive chemotherapy, that is large-scale distribution of anthelminthic drugs to populations at risk, as the main intervention strategy against liver flukes [12], [13]. If the prevalence in a district is >20%, universal treatment of all individuals in the district is recommended once a year. If the prevalence is <20%, the recommendation is either a universal treatment every second year, or targeted treatment once a year of all those who report habitually eating raw fish [12]. A similar intervention strategy is used in the well-established programs to control other helminth infections like schistosomiasis, geo-helminth infections, onchocerciasis and filariasis [14]. The effectiveness of preventive chemotherapy on *C. sinensis* has been examined in Chinese studies [15]. Choi et al. published a large study with 14139 people, evaluating different regimes of universal treatment or selective treatment over a three-year period. The different regimes gave a reduction in prevalence of *C. sinensis* between 34.2–93.9% after one year following treatment and 72.7–95.6% after three years [16]. However, there are few studies from other endemic areas evaluating the effectiveness of preventive chemotherapy, and the information originates mostly from studies done more than 20 years ago of which several were conducted within the same study population [17]–[22]. A study from 1988 from a village in Thailand with a *O. viverrini* prevalence of around 90% showed that 88.4% of the people was egg negative two weeks after praziquantel treatment (n = 704). However, 87.7% of those negative after two weeks became reinfected within a year [23]. Nissen et al. found that half of the North Vietnamese farm dogs that had been given a single dose of praziquantel were reinfected with FZT within three months [24]. Hence they could not recommend anthelmintic treatment as the only intervention to control FZT in dogs.

The use of preventive chemotherapy as an intervention to control FZT in Vietnam started in 2006 and has gradually increased since then. In 2011 such treatment was given to 128837 people, amounting to 532053 tablets of praziquantel (NIMPE, annual report 2011). Preventive chemotherapy is here given as selective chemotherapy to people confirming that they eat raw fish. Despite the large number of treatments, the effectiveness of preventive chemotherapy in Vietnam has not been thoroughly assessed. A pilot study from Nam Dinh province involving 21 FZT egg-positive people showed a prevalence of 29% and 71% six days and five weeks after having received praziquantel treatment, respectively [25]. The authors refer the infections as *C. sinensis*, but the species distribution in that province makes it more likely to be a mix of *C. sinensis* and MIF [8].

In the present study, the rate of reinfection with FZT was assessed after applying preventive chemotherapy for the first time in a cohort of people confirmed having eating raw fish in two communes in Northern Vietnam.

## Methods

### Ethics

Informed, written consent was obtained from participants aged 16 years or older. In accordance with Vietnamese regulations, guardians signed for minors under the age of 16 years. The study protocol was approved by the Ethics Committee at the National Institute of Malariology, Parasitology and Entomology (NIMPE), Hanoi. The present study was part of the regular preventive chemotherapy program for FZT infections in the area. Hence, refusing to participate in the study did not prevent people from receiving preventive chemotherapy. Information about the disease and treatment was given initially according to standard praxis for the control program. Appropriate treatment was offered if other helminth eggs were found by microscopy. The practice of the present study implied intensified treatment compared with the normal setting for preventive therapy program, in which there is no testing after preventive chemotherapy and therefore no further treatment.

### Study population and design

The study was conducted between March 2011 and June 2012 in Nghia Hong commune, Nghia Hung district and Hai Hoa commune, Hai Hau district, both located in Nam Dinh province, in Northern Vietnam. The communes have approximately 7800 and 8700 inhabitants, respectively. Neither of the two communes had been part of the regional program for preventive chemotherapy prior to the study, and neither of the communes had been part of surveys in which people could have received anthelmintic treatment for the last three years prior to the study. Before the study, both communes were assessed by NIMPE, Hanoi, and found to be eligible for the regional program for preventive chemotherapy for FZT infections. Potential participants were approached following the standard procedure of this program. A village health worker visited the households and asked who had ever eaten raw fish. People confirming eating raw fish were invited to participate in preventive chemotherapy if they were not pregnant or suffering from liver or kidney disease, serious hypertension or acute stomach symptoms. Those selected received 50 mg/kg praziquantel, split in two doses on the same day. A randomly selected subgroup of those eligible for preventive chemotherapy were invited into the study and asked to deliver a stool sample three weeks prior to and two, 16, 29 and 60 weeks after the preventive chemotherapy was given. Each stool sample was examined with Kato-Katz technique, using two slides for each sample [26]. The number of eggs per gram stool (epg) was recorded.

**Figure 1:**
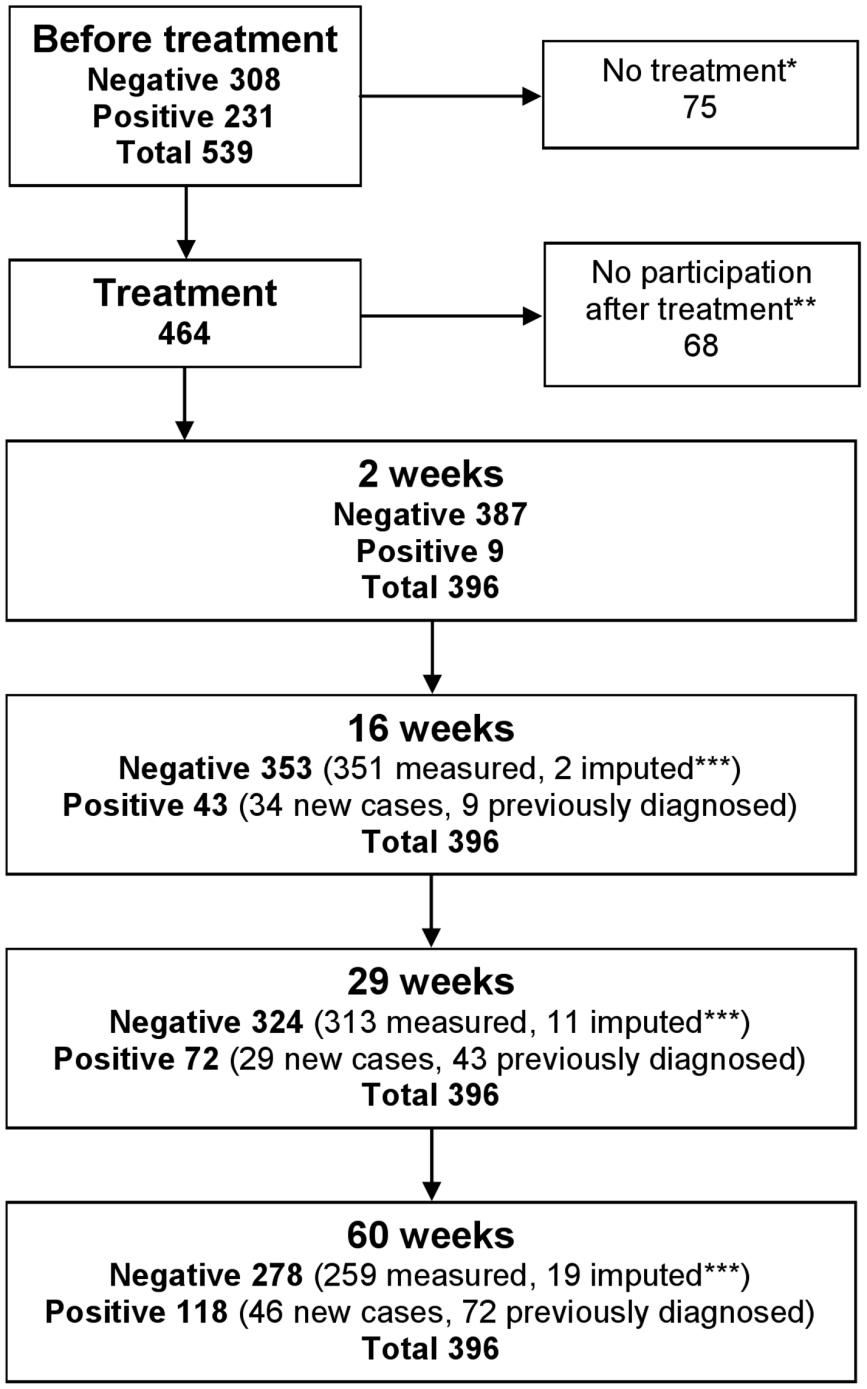
Study design and cumulative absolute numbers for prevalence of fishborne zoonotic trematode eggs before preventive chemotherapy treatment and two, 16, 29 and 60 weeks after preventive chemotherapy. *Did not receive treatment in time to be included in the study. **Did not deliver any stool samples after treatment and were excluded from further analysis. *** Missing stool samples after preventive chemotherapy were handled with imputation, i.e. a missing stool sample was regarded as negative if the previous test in the timeline was negative.

A total of 539 people delivered a stool sample prior to preventive chemotherapy. From these, 75 people did not receive preventive chemotherapy and 68 failed to deliver any stool samples after preventive chemotherapy, hence, they were excluded from the follow up study. Overall, 396 people (73.5%) had sufficient data to be included in the final analysis. Those found positive by microscopy after preventive chemotherapy received a treatment with praziquantel and were not examined further, but regarded as positive for the remaining of the study. Hence the reported figures for reinfection are cumulative. Missing stool samples after preventive chemotherapy were handled with imputation, i.e. a missing stool sample at time 16 weeks, 29 weeks and/or 60 weeks were regarded as negative if the previous test in the timeline was negative. The study design and the absolute figures of test results are presented in Figure 1.

### Statistical analysis

Descriptive measures of prevalence and intensity of infection were performed with IBM SPSS Statistics (Version 20.0. Armonk, NY: IBM Corp). Intensity of infection is presented by median value, not mean, due to kurtosis of distribution. Confidence intervals around medians were created using bootstrapping method. The cumulative prevalence of infection is presented in a bar diagram using Microsoft Excel 2007 (Redmond, Washington). The findings in this simple presentation were confirmed in an repeated measure analysis of variance (SPSS). Confidence intervals around the prevalence were calculated in SPSS using Wilson's method without continuity correction. Multiple logistic regression (SPSS) were used for analysis of risk factors for reinfection. Reinfection among those negative and those positive before treatment is presented in a bar diagram using MicrosoftExcel 2007.

## Results

Study subjects included 242 men (61%) with a median age of 50 years (range 8–75) and 154 women (39%) with a median age of 47 years (range 15–75).

**Figure 2:**
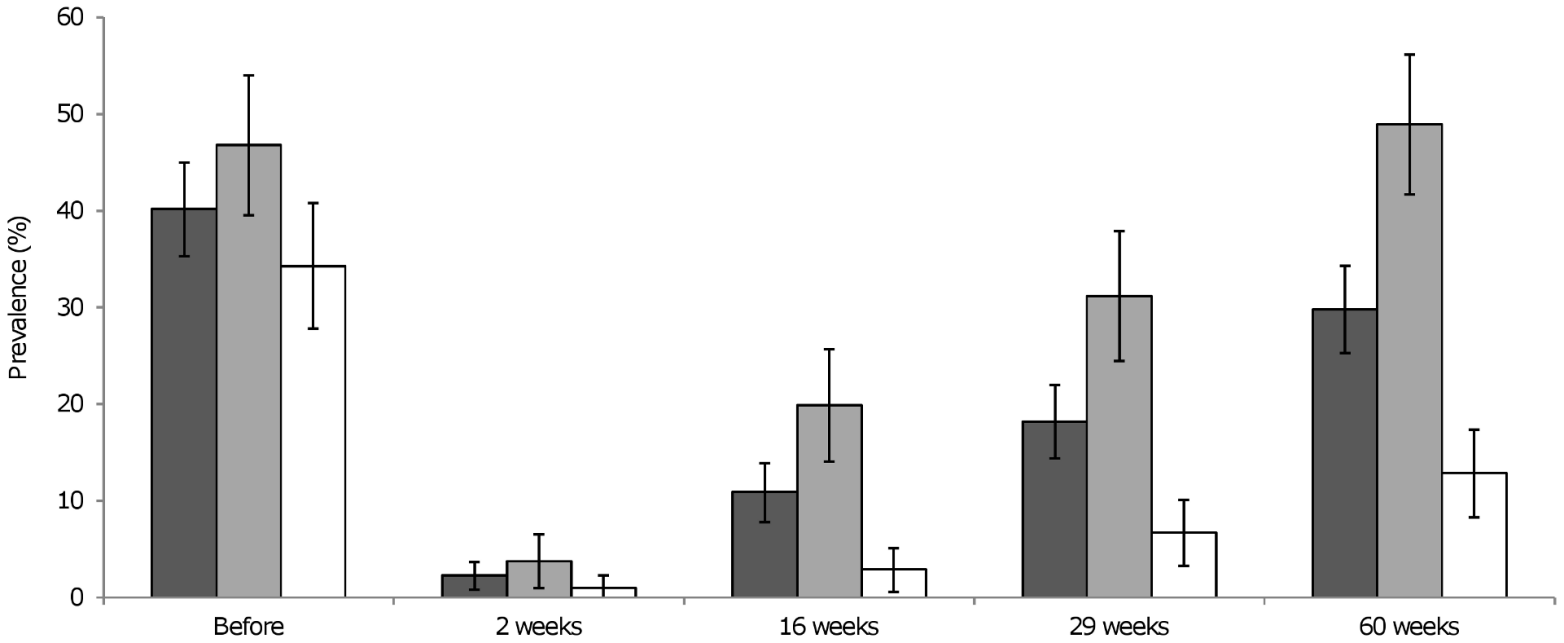
Prevalence of fishborne zoonotic trematode eggs in a cohort of 396 people before preventive chemotherapy, and cumulative prevalence two, 16, 29 and 60 weeks after preventive chemotherapy. Dark grey: Both communes combined. Medium grey: Nghia Hong commune (n = 186). White: Hai Hoa commune (n = 210). Error bars are 95% confidence intervals.

As seen in Figure 2, the prevalence of small trematode eggs in stool before preventive chemotherapy for the two sites combined were 40.2% (95% confidence interval (CI) of 35.3–45.0). The prevalence two, 16, 29 and 60 weeks after preventive chemotherapy were 2.3% (CI 0.8–3.7), 10.9% (CI 7.8–13.9), 18.2% (CI 14.4–22.0) and 29.8% (CI 25.3–34.3), respectively. The increase in prevalence after preventive chemotherapy is significant between all time points, as seen by the repeated measure ANOVA analysis (table 1). The FZT prevalence among all the 539 who delivered stool sample before preventive chemotherapy was 42.9% (CI 38.7–47.0), not different from the group of 396 constituting the cohort followed up.

**Table 1:** Cumulative prevalence and change in cumulative prevalence.

	Cumulative prevalence %	Change in cumulative prevalence* % (95% CI)
Before	40.2	-
2 weeks	2.3	−37.9 (−31.0: −44.8)
16 weeks	10.9	8.6 (4.6: 12.6)
29 weeks	18.2	7.3 (3.6: 11.0)
60 weeks	29.8	11.6 (7.1: 16.2)

Cumulative prevalence and change in cumulative prevalence before and two, 16, 29 and 60 weeks after preventive chemotherapy. Change in cumulative prevalence with 95% confidence intervals. *Repeated measure ANOVA.

Results from the two study sites differed when analyzed separately (Fig. 2). Nghia Hong commune had a more rapid increase in FZT prevalence after chemotherapy. The prevalence after 60 weeks in Nghia Hong commune was the same as before preventive chemotherapy. In Hai Hoa commune, the FZT prevalence at the end of the study was less than half of that before preventive chemotherapy, and significantly lower than in Nghia Hong commune, as seen by the confidence intervals. The overall infection intensity, measured as median egg per gram stool, was low throughout the study (Table 2). However, the range between the highest and lowest epg was high, even as soon as 16 weeks after preventive chemotherapy.

**Table 2:** Infection intensity.

	Before	W2	W16	W29	W60
Median	72	48	36	72	60
CI median	60 : 84	24 : 60	24 : 48	36 : 120	36 : 96
Minimum	12	12	12	12	12
Maximum	3312	72	2628	828	1248
N (positive)	159	9	34	34	68

Number of fishborne zoonotic trematode eggs per gram stool in a cohort of 396 people before and two, 16, 29 and 60 weeks (Before, W2, W16, W29, W60) after preventive chemotherapy. Figures shown are medians with 95% confidence intervals, minimum and maximum value. Number of egg positive persons at each time point is also shown.

It is noteworthy that 76/118 (65%) of those people egg-positive after 60 weeks were also positive before treatment, suggesting a relative high degree of reinfection in these people. When analyzing gender, age and previous infection as risk factors for reinfection at 60 weeks of follow up, we see in the single logistic regression model that significantly more men than women become reinfected. Increasing age is also associated with reinfection, as is previous infection. However, in the multiple logistic regression model only previous infection remains associated with reinfection, indicating confounding. Before treatment, a significantly higher proportion of the participating men than the participating women were infected (53% versus 20%, p<0.001). A similar pattern was found 60 weeks after treatment, with 36% of the men and 20% of the women being infected (p<0.001).

## Discussion

**Figure 3:**
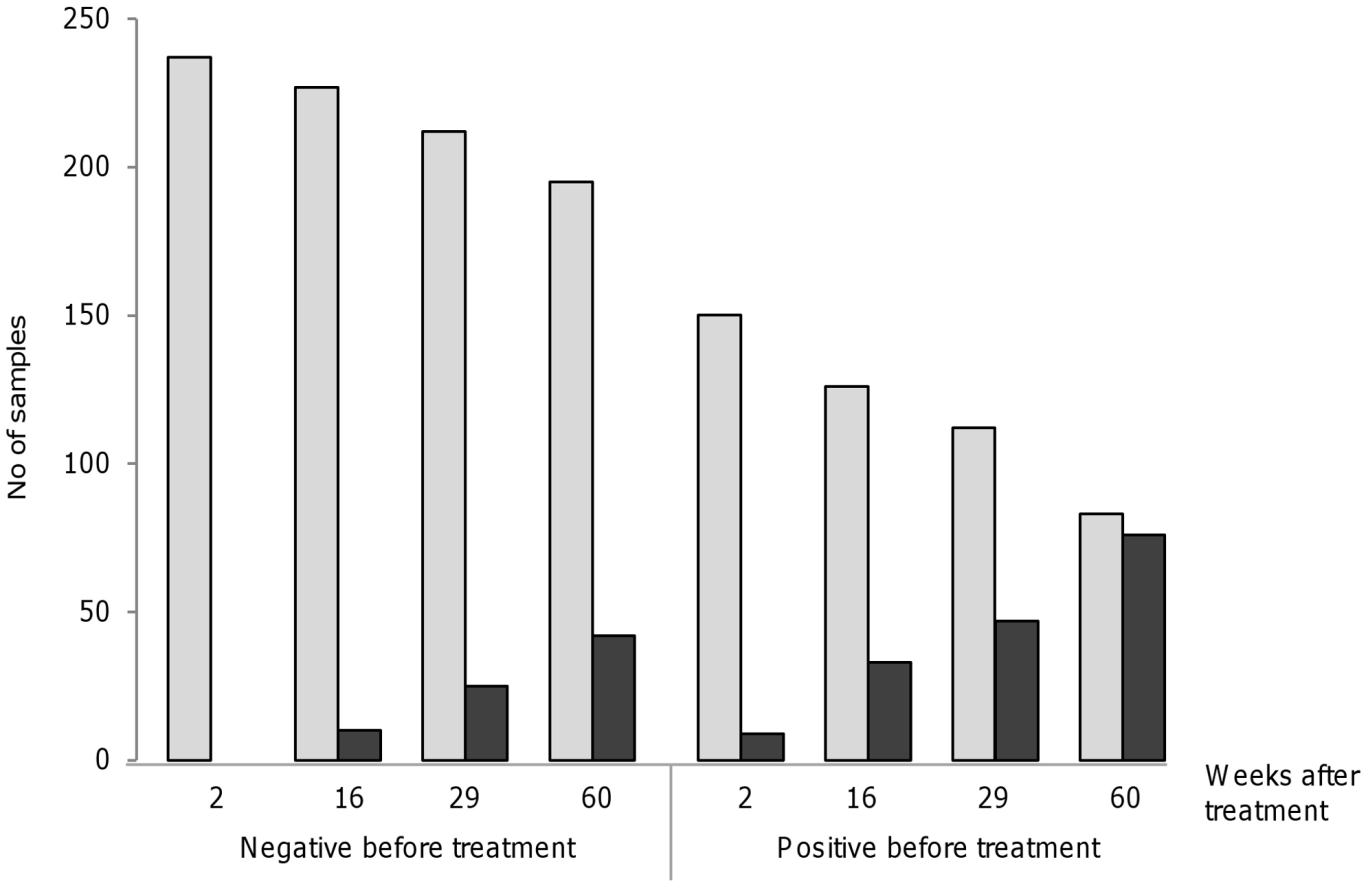
Cumulative reinfection rate two, 16, 29 and 60 weeks after treatment (preventive chemotherapy). The group with negative sample before treatment to the left and the group with positive sample before treatment to the right. Dark grey bars are cumulative positive samples. Light grey bars are negative samples. Missing stool samples after treatment were handled with imputation, i.e. a missing stool sample was regarded as negative if the previous test in the timeline was negative.

In the present study, we found a high reinfection rate of FZT within one year following treatment with praziquantel. The true reinfection rate might have been even higher as we applied a conservative analytical approach, categorizing a person as non-infected in case of non-participation during follow up. Also, as infected persons were treated during follow up, their contribution to FZT transmission was reduced. Our estimate of reinfection is thus conservative. We found a difference in degree of reinfection between the two communes. Whether this is due to a difference in the frequency of eating raw fish, intensity of FZT metacercariae in fish or due to other reason is not known. Figure 3 shows an increased rate of reinfection among those positive before treatment. This is confirmed in the multiple logistic regression model (Table 3). In this model we found that previous infection (representing risk behavior) was a significant risk factor for reinfection, whereas male gender and increasing age were not. This indicates that the seemingly close association between male gender, increasing age and reinfection we observed (crude numbers), is possibly confounded by risk behavior of eating raw fish, a tradition mostly common among men [27].

**Table 3:** Gender, age and infection before treatment as risk factors.

	Crude Odds Ratio (95% CI)	Adjusted Odds Ratio (95% CI)
Male gender (female ref.)	2.4 (1.5 : 3.8)	1.53 (0.91 : 2.57)
Age	1.03 (1.01 : 1.04)	1.02 (0.99 : 1.03)
Infected before treatment	4.24 (2.7 : 6.7)	3.52 (2.18 : 5.69)

Gender, age and infection before treatment (preventive chemotherapy) as risk factors of cumulative infection 60 weeks after treatment. Adjusted model includes gender, age and infection before treatment. Logistic regression.

FZT infections in Northern Vietnam are a mix of *C. sinensis* and MIF. *C. sinensis* seems to be much less common in fish than in humans. However, only very few studies have investigated the species distribution of FZT in humans. Phan et al. [28] examined 1543 fish from Nam Dinh province, Northern Vietnam, of which more than half were infected with FZT metacercariae. Only a single fish contained *C. sinensis* metacercariae. In humans, Trung et al. [8] identified the species of adult worms expulsed from 615 egg-positive people from Nam Dinh province. All the people had MIF, and 51.5% had in addition *C. sinensis*. We do not yet have an explanation for the seemingly different species distribution in fish and humans. One possible explanation is that it is a relatively rare event for a human to acquire an infection with *C. sinensis* compared to a MIF infection, but once acquired, *C. sinensis* infections are long lived, 26 years has been described [29], [30]. Annual preventive chemotherapy may then decrease the proportion of *C. sinensis* infections in humans, as by far most of the reinfection will be due to MIF. Since *C. sinensis* probably have a higher morbidity and mortality than MIF, such a shift in species distribution may be an argument for preventive chemotherapy even if the reinfection rate is high. However, we do not know how preventive chemotherapy influences the species distribution, and such a shift has yet to be shown.

Some aspects in the design of the Vietnamese control program may become a challenge. In Vietnam, the preventive chemotherapeutic dose of 50 mg/kg is split into two doses to minimize side-effects. This makes it impossible to have direct observed therapy. In the present study, the FZT prevalence dropped from 40.2% before preventive chemotherapy to 2.3% two weeks after. This drop is a combination of praziquantel efficacy and compliance. Hence, the compliance in our study was excellent, but compliance may become a problem especially if preventive chemotherapy is continued for several years and without direct observed therapy [31]. Vietnam has also found it cost-effective to perform preventive chemotherapy as selective chemotherapy by only treating those who confirm that they have ever eaten raw fish [32], [33]. However, Phan et al. [27] found in a study involving 180 persons that 38.5% of those confirming eating raw fish were egg positive on microscopy, but also 25.8% of those who denied eating raw fish. A possible explanation for this may be that especially women often have the perception that they do not eat raw fish, but during food preparation they taste the raw fish dishes which is a significant risk [34]. Additionally, cross-contamination might occur because bowls with sauces are typically shared at meals, i.e. by people dipping pieces of raw fish and those dipping raw vegetables only. If this is to be confirmed, the current strategy on how to identify people to receive treatment may have to change.WHO promotes the use of preventive chemotherapy to control FZT. This is in accordance with the control strategy for several other helminths, like schistosomiasis, geo-helminth infections, onchocerciasis and filariasis. Even though preventive chemotherapy has shown to be efficient in reducing the prevalence and infection intensity of these infections, rapid reinfection after treatment is also well documented [35]. However, in FZT the the scarcity of studies provides little scientific support for this strategy. None of the mentioned helminth infections, with the exception of *Schistosoma japonicum*, are zoonoses, and hence have a quite different transmission than FZT. In the FZT lifecycle, both humans and domestic as well as wild animals pass eggs to the environment. The presence of infected snails and introduction of infected fish into the freshwater environment are also important in the transmission of FZT [36]–[38]. It is difficult to evaluate which factor makes the largest contribution to the transmission, and it is likely that this may vary according to FZT species, geographical area and type of aquatic environment. Further, it should be noted we do not know to what extend people infected with FZT obtained such infection from the consumption of raw dishes of fish originating from aquaculture or wild-caught. Nguyen et al. [9], [10] found that dogs, cats and pigs are important sources of FZT eggs, in particular minute intestinal fluke eggs, to the environment in two endemic areas in Northern Vietnam. Cats and dogs deposit their stool freely, while pig manure is used as fertilizer for fish ponds and rice fields. As humans are only one of several reservoirs that contribute to the transmission, decreasing the egg output through preventive chemotherapy of humans may not have a lasting effect on reducing transmission and risk of FZT infection.

We focused on reinfection in the first year after preventive chemotherapy. If repeated preventive chemotherapy decreases the prevalence of infections, it may have an effect on morbidity. However, if the human contribution to the lifecycle of FZT is only minor, transmission is not sufficiently affected, and it may be difficult to terminate the use of preventive chemotherapy after a limited number of years. Control of FZT, being a zoonosis, may in the long run benefit from an increased focus on a more integrated approach, as experienced in Thailand [4]. An integrated approach to reduce snail population and egg contamination has also been studied in Vietnamese fish nurseries by Clausen et al. [37]. Two intervention groups were studied; one farm management group with control of snail vectors and fecal pollution of pond, and one group with drug treatment of humans and animals. It was found that the intensity of metacercariae in fish was reduced with 91.7% in the farm management group. Even though veterinary public health has been mentioned by WHO as an intervention to overcome the Neglected Tropical Diseases, preventive chemotherapy has so far been the dominating pillar in the attempt to control FZT [12], [14]. ‘One Health’ is a concept focusing on integration between human and veterinary medicine. This may be a useful concept if interventions such as discouraging the use of raw fish for animal feed, discouraging the use of untreated pig manure as fertilizer in ponds and regular anthelmintic treatment of roaming domestic animals are to be included in the control of FZT [10]. FZT has, apart from being a zoonosis, many similarities with other Neglected Tropical Diseases which coexist in the same population. A recent policy paper argues for multi-disease, multi-sectoral synergistic control interventions for helminth infections in the Western Pacific region [39].

In conclusion, we question if preventive chemotherapy alone is sufficient to control the FZT and suggest that a more integrated approach including One Health and improved aquaculture farm management should be explored.

## Supporting Information

Checklist S1 STROBE checklist.
(DOCX)
