# Supplementary material for: High Reinfection Rate after Preventive Chemotherapy for Fishborne Zoonotic Trematodes in Vietnam
Source: PLoS Negl Trop Dis. 2014 Jun 19;8(6):e2958. doi: 10.1371/journal.pntd.0002958 (PMC4063704; doi:10.1371/journal.pntd.0002958)
Supplement: Checklist S1 — STROBE checklist. (DOCX) [file pntd.0002958.s001.docx]

STROBE Statement—Checklist of items that should be included in reports of ***cohort studies***

|  | Item No | Recommendation |
| --- | --- | --- |
| **Title and abstract** | 1 | (*a*) Indicate the study’s design with a commonly used term in the title or the abstract ***Abstract;Methodology*** |
|  |  | (*b*) Provide in the abstract an informative and balanced summary of what was done and what was found ***Abstract*** |
| Introduction | | |
| Background/rationale | 2 | Explain the scientific background and rationale for the investigation being reported ***Introduction*** |
| Objectives | 3 | State specific objectives, including any prespecified hypotheses ***Introduction, last part*** |
| Methods | | |
| Study design | 4 | Present key elements of study design early in the paper ***Methods; Study population and design*** |
| Setting | 5 | Describe the setting, locations, and relevant dates, including periods of recruitment, exposure, follow-up, and data collection ***Methods; Study population and design*** |
| Participants | 6 | (*a*) Give the eligibility criteria, and the sources and methods of selection of participants. Describe methods of follow-up ***Methods; Study population and design*** |
|  |  | (*b*) For matched studies, give matching criteria and number of exposed and unexposed ***N.A.*** |
| Variables | 7 | Clearly define all outcomes, exposures, predictors, potential confounders, and effect modifiers. Give diagnostic criteria, if applicable ***Methods; Study population and design. Results, first part*** |
| Data sources/ measurement | 8* | For each variable of interest, give sources of data and details of methods of assessment (measurement). Describe comparability of assessment methods if there is more than one group. ***Methods; Study population and design, middle part*** |
| Bias | 9 | Describe any efforts to address potential sources of bias ***Methods; Study population and design, middle part. Results, middle part*** |
| Study size | 10 | Explain how the study size was arrived at a) |
| Quantitative variables | 11 | Explain how quantitative variables were handled in the analyses. If applicable, describe which groupings were chosen and why ***Methods; Statistical analysis*** |
| Statistical methods | 12 | (*a*) Describe all statistical methods, including those used to control for confounding ***Methods; Statistical analysis*** |
|  |  | (*b*) Describe any methods used to examine subgroups and interactions ***NA*** |
|  |  | (*c*) Explain how missing data were addressed ***Methods; Study population and design, last part.*** |
|  |  | (*d*) If applicable, explain how loss to follow-up was addressed ***Methods; Study population and design, last part.*** |
|  |  | (*e*) Describe any sensitivity analyses ***NA*** |
| Results | | |
| Participants | 13* | (a) Report numbers of individuals at each stage of study—eg numbers potentially eligible, examined for eligibility, confirmed eligible, included in the study, completing follow-up, and analysed ***Fig 1. Methods; Study population and design, last part.*** |
|  |  | (b) Give reasons for non-participation at each stage ***Fig 1. Methods; Study population and design, last part.*** |
|  |  | (c) Consider use of a flow diagram ***Fig 1*** |
| Descriptive data | 14* | (a) Give characteristics of study participants (eg demographic, clinical, social) and information on exposures and potential confounders. ***Results, first part. Fig 2*** |
|  |  | (b) Indicate number of participants with missing data for each variable of interest ***Fig 1*** |
|  |  | (c) Summarise follow-up time (eg, average and total amount) ***Fig 1. Results, first part.*** |
| Outcome data | 15* | Report numbers of outcome events or summary measures over time ***Fig 1-3*** |
| Main results | 16 | (*a*) Give unadjusted estimates and, if applicable, confounder-adjusted estimates and their precision (eg, 95% confidence interval). Make clear which confounders were adjusted for and why they were included ***Fig 2. Tables 1-3*** |
|  |  | (*b*) Report category boundaries when continuous variables were categorized ***NA*** |
|  |  | (*c*) If relevant, consider translating estimates of relative risk into absolute risk for a meaningful time period ***NA*** |
| Other analyses | 17 | Report other analyses done—eg analyses of subgroups and interactions, and sensitivity analyses ***NA*** |
| Discussion | | |
| Key results | 18 | Summarise key results with reference to study objectives ***Discussion, first part*** |
| Limitations | 19 | Discuss limitations of the study, taking into account sources of potential bias or imprecision. Discuss both direction and magnitude of any potential bias ***Discussion, first, middle and last part. Introduction, first part*** |
| Interpretation | 20 | Give a cautious overall interpretation of results considering objectives, limitations, multiplicity of analyses, results from similar studies, and other relevant evidence. ***Discussion, first, middle and last part.*** |
| Generalisability | 21 | Discuss the generalisability (external validity) of the study results ***Discussion, middle and last part.*** |
| Other information | | |
| Funding | 22 | Give the source of funding and the role of the funders for the present study and, if applicable, for the original study on which the present article is based ***b)*** |

*Give information separately for exposed and unexposed groups.

**Note:** An Explanation and Elaboration article discusses each checklist item and gives methodological background and published examples of transparent reporting. The STROBE checklist is best used in conjunction with this article (freely available on the Web sites of PLoS Medicine at http://www.plosmedicine.org/, Annals of Internal Medicine at http://www.annals.org/, and Epidemiology at http://www.epidem.com/). Information on the STROBE Initiative is available at <http://www.strobe-statement.org>.

***a) Sample size:*** ***From previous prevalence studies it is known that the prevalence of infection in a high-risk population is approximately 50% (Dung, personal communication). The praziquantel treatment has a high efficacy and we will expect the prevalence of infection in the high-risk population receiving preventive chemotherapy to drop to a level of about 3%. The follow up during the next year will show at what speed reinfection occur. The minimum increase that we wish to detect is an increase up to a prevalence of 10%. From previous studies with fishborne trematodes we expect that the actual increase will be higher (Upatham et al., 1988; Upatham & Viyanant, 2003; Nithikthkul et al., 2004). To show an increase from 3% to 10%, with a risk of making a type 1 error of 0.05, and a power of 0.80 to detect a difference, we need a sample size of 200 (ref: Statistical calculator, Department of statistics, University of British Columbia). We think it necessary to do the study in two communes, as there may be environmental, human and social factors we cannot control for, which makes the study vulnerable if it is done in only one commune.***

***We therefore plan for two study populations of 200 persons each, altogether 400 persons.***

***b) Funding:*** ***Danish International Development Assistance (Danida) through the ‘‘Fishborne Zoonotic Parasites in Vietnam’’ (FIBOZOPA)-project( project no 104 Dan-8-L-717-LIFE2) have funded the study. The funders had no role in study design, data collection and analysis, decision to publish, or preparation of the manuscript.***
